# Understanding the Interaction of Röntgen Radiation Employed in Computed Tomography/Cone Beam Computed Tomography Investigations of the Oral Cavity by Means of Surface-Enhanced Raman Spectroscopy Analysis of Saliva

**DOI:** 10.3390/s24248021

**Published:** 2024-12-16

**Authors:** Rareș-Mario Borșa, Valentin Toma, Melania-Teodora Nășcuțiu, Anca Onaciu, Ioana-Maria Colceriu-Șimon, Grigore Băciuț, Simion Bran, Cristian-Mihail Dinu, Florin Onișor, Gabriel Armencea, Carina Culic, Mihaela-Carmen Hedeșiu, Rareș-Ionuț Știufiuc, Mihaela-Felicia Băciuț

**Affiliations:** 1 Dental Medicine Faculty, “Iuliu Hatieganu” University of Medicine and Pharmacy, Pasteur 4, 400349 Cluj-Napoca, Romania; rares.mari.borsa@elearn.umfcluj.ro (R.-M.B.); melania.teod.nascutiu@elearn.umfcluj.ro (M.-T.N.); gbaciut@umfcluj.ro (G.B.); dr_brans@umfcluj.ro (S.B.); cristian.dinu@umfcluj.ro (C.-M.D.); florin.onisor@umfcluj.ro (F.O.); armencea.gabriel@umfcluj.ro (G.A.); mbaciut@umfcluj.ro (M.-F.B.); 2 Department of Maxillofacial Surgery and Implantology, “Iuliu Hațieganu” University of Medicine and Pharmacy, Cardinal Iuliu Hossu 37, 400029 Cluj-Napoca, Romania; 3 Department of Pharmaceutical Physics & Biophysics, Faculty of Pharmacy, “Iuliu Hatieganu” University of Medicine and Pharmacy, Louis Pasteur 6, 400349 Cluj-Napoca, Romania; anca.onaciu@umfcluj.ro; 4 Department of Prosthetic Dentistry and Dental Materials, Division Dental Propaedeutics, Aesthetics, Dental Medicine Faculty, “Iuliu Hatieganu” University of Medicine and Pharmacy, Clinicilor 32, 400001 Cluj-Napoca, Romania; 5 Department of NanoBioPhysics, Institute of Medical Research and Life Sciences—MEDFUTURE, “Iuliu Haţieganu” University of Medicine and Pharmacy, Louis Pasteur 4-6, 400349 Cluj-Napoca, Romania; valentin.toma@umfcluj.ro; 6 Department of Conservative Odontology, Division Orthodontics and Dental-Facial Orthopedics, “Iuliu Hatieganu” University of Medicine and Pharmacy, Avram Iancu 31, 400089 Cluj-Napoca, Romania; simon.ioana@umfcluj.ro; 7 County Emergency Hospital Cluj, Clinicilor 3-5, 400006 Cluj-Napoca, Romania; mhedesiu@umfcluj.ro; 8 Department of Conservative Odontology, Division Odontology, Endodontics, Cariology, Oral Pathology, “Iuliu Hatieganu” University of Medicine and Pharmacy, Moților 33, 400089 Cluj-Napoca, Romania; culic@elearn.umfcluj.ro; 9 Department of Oral Radiology, “Iuliu Hatieganu” University of Medicine and Pharmacy, Avram Iancu 31, 400089 Cluj-Napoca, Romania; 10 Nanotechnology Laboratory, TRANSCEND Research Center, Regional Institute of Oncology, General Henri Mathias Berthelot 2-4, 700483 Iaşi, Romania

**Keywords:** saliva, X-rays, CBCT, CT, SERS, biomarkers

## Abstract

The use of Raman spectroscopy, particularly surface-enhanced Raman spectroscopy (SERS), offers a powerful tool for analyzing biochemical changes in biofluids. This study aims to assess the modifications occurring in saliva collected from patients before and after exposure to cone beam computed tomography (CBCT) and computed tomography (CT) imaging. SERS analysis revealed significantly amplified spectra in post-imaging samples compared to pre-imaging samples, with pronounced intensification of thiocyanate and opiorphin bands, which, together with proteins, dominated the spectra. The changes were more pronounced in the case of CT as compared to CBCT, probably due to the use of a high radiation dose in the case of the first-mentioned technique. These findings underscore the impact of CBCT and CT on salivary composition, highlighting the relevance of SERS as a sensitive method for detecting subtle molecular changes in biofluids post-radiation exposure. This study’s results emphasize the importance of monitoring biochemical markers in patients undergoing diagnostic imaging to better understand the systemic effects of ionizing radiation.

## 1. Introduction

X-rays represent a form of high-energy electromagnetic radiation, belonging to the category of ionizing radiation alongside gamma rays. These rays are also known as Röntgen radiation, named in honor of the German scientist Conrad Wilhelm Röntgen, who discovered them in 1895 [1].

Unlike visible light radiation, X-rays have a significantly shorter wavelength. They are not electrically charged, which allows them to remain unaffected by electric or magnetic fields. For these reasons, X-rays have the ability to penetrate various thin opaque materials, such as muscle tissues, but not bone tissues, which have diametrically opposite characteristics [1]. X-rays are widely used in medicine, particularly in the fields of radiodiagnosis, radiotherapy, fluoroscopy, and sterilization.

In dentistry, imaging methods often represent the primary means by which practitioners arrive at a diagnosis. Over time, thanks to scientific progress, the development and refinement of complementary diagnostic techniques have been made possible. The study of the characteristics of conventional computed tomography (CT) alongside cone beam computed tomography (CBCT) is a relevant part of this study.

The CT technique was patented by Godfrey Hounsfield in 1972, based on the mathematical image reconstruction developed by Alan Cormack in the 1950s and 1960s [2]. X-rays are emitted in the form of a collimated, fan-shaped beam, which then passes through the entity being analyzed and is received by scintillation detectors. These detectors quantify the number of emerging photons (X-rays) from the entity under investigation. Thus, by rotating the tube circumferentially around the object, cross-sectional images of the structures of interest are formed based on the reconstruction of the internal structure from multiple Röntgen ray projections [3].

CBCT is a significant technological advancement in maxillofacial imaging, superior to panoramic radiography. CBCT uses a two-dimensional digital detector array and a three-dimensional X-ray beam with circular collimation, forming a cone-shaped beam. This allows for the rapid acquisition of volumetric data from a single rotational scan of the gantry, saving costs compared to traditional CT [4].

CBCT, initially developed for angiography in the 1980s and commercially introduced for dental and maxillofacial use in the late 1990s and early 2000s, enables 3D imaging for the evaluation of the dento-maxillofacial complex, facilitating dental diagnosis [5].

The optimization of diagnostic formulation, planning, and personalization of dental treatments are consequences of continuous innovation in health research, along with a better understanding of etiopathogenetic mechanisms.

Ultrasensitive vibrational spectroscopy is a promising research technique in the fascinating field of dental medicine. Among its multiple applications are the quantitative and qualitative chemical analysis of liquid/solid biopsies and dental materials [6,7,8].

Raman spectroscopy is a vibrational spectroscopic technique based on the inelastic scattering of photons from an excitatory monochromatic laser radiation. With the help of this technique, the molecules present in the analyzed samples (molecular fingerprint) can be identified. One of the major disadvantages of this method is the low sensitivity when analyzing biomolecules with low concentrations [9].

Surface-enhanced Raman spectroscopy (SERS) uses substrates that possess plasmonic properties. These substrates allow the amplification of the Raman vibrational spectra of the analyte molecules. In this sense, substrates synthesized on the basis of colloidal silver or gold nanoparticles are a highly used class in biofluid analysis. The spectra recorded with these plasmon substrates can be used to develop new diagnostic strategies for various diseases belonging to the broader class of liquid biopsy methods [10,11,12].

At the same time, this method allows the detection of analytes in low concentrations by amplifying the general electromagnetic fields by the excitation of plasmons located on the surface of noble metal nanoparticles (gold, silver). In recent years, the SERS analysis technique has evolved tremendously, and currently we can even discuss the use of SERS in single-molecule detection. Its advantages include high sensitivity, real-time spectral analysis, as well as the possibility of its use in point-of-care applications [9].

Over time, various studies have been carried out on the saliva of human subjects in order to develop methods for the early detection of oral and systemic pathologies.

Sialometry (quantitative salivary analysis) and sialochemistry (qualitative salivary analysis), combined with Raman spectroscopy, have been used to monitor the subjects’ overall health [13].

The use of saliva as an investigation method is currently one of the most fascinating diagnostic techniques, because although there is an impressive variability in the quantity and quality of patients’ salivary secretion, the composition of salivary products can still play a role in diagnosing pathologies. There are many studies related to the use of saliva samples for detecting oral cancer [6,7,14], Sjögren’s syndrome [15], SARS-CoV-2 infection [16,17], oxidative stress markers [18], and other biomarkers by means of SERS.

Saliva, used as a diagnostic biofluid, has multiple benefits: it is easier to harvest than any other biofluid and allows for a quick and minimally invasive collection that can be carried out without prior special training. The risk of infection for the donor is negligible, and the risk assumed by the handler is lower compared to blood. The possibility of supervising the collection of saliva samples significantly reduces the chance of them being compromised, which would justify their preference in forensic or forensic investigations [13].

In this study, SERS spectroscopy has been employed for the analysis of the salivary composition modifications before and after patient exposure to X-rays. This technique has enormous benefits, like the possibility of comparing samples from the same donor. As a consequence, some relevant salivary biomarkers and their diagnostic potential were investigated.

## 2. Materials and Methods

### 2.1. Saliva Sample Collection and Processing

The present study was carried out in accordance with the legislative aspects regulated by the Ethics Commission for Scientific Research of the “Iuliu Haţieganu” University of Medicine and Pharmacy of Cluj-Napoca, respectively, at the Cluj County Emergency Clinical Hospital.

A total of 15 patients who underwent a CT investigation (at the Radiology Clinic of Cluj County Emergency Clinical Hospital) and 15 patients who underwent a CBCT imaging investigation (at the Oral and Maxillofacial Surgery Clinic of Cluj County Emergency Clinical Hospital) between November 2023 and March 2024 were included in this study.

The following inclusion criteria were respected: adult subjects with or without oral and systemic pathologies who needed complementary radiological examinations such as CT and CBCT. Patients following orthodontic treatment, possessing metallic dentures, along with those from whom insufficient saliva samples were collected and those from whom saliva samples mixed with blood and significant food detritus were collected, were excluded. Written informed consent was signed by every patient.

From each patient, a saliva sample was collected before exposure (pre-exposure) and 30 min after performing the CT or CBCT imaging technique (post-exposure). Patients were asked to donate a sample of 2 mL of saliva before and after the investigation. The samples were subjected to the centrifugation process for 1 min at 4 °C at 15,000× *g*. After centrifugation, the supernatant was removed from the samples and transferred to 0.5 mL Eppendorf tubes and stored in the freezer at −80 °C until the time of SERS analysis.

### 2.2. SERS Substrate Preparation

The synthesis of Ag colloids was performed according to the developed Leopold–Lendl method [19]. The characteristics of the colloids thus obtained were evaluated using the following methods: UV-Vis absorption spectroscopy and transmission electron microscopy (TEM).

The obtained colloids were concentrated 10 times using the tangential flow filtration (TFF) method using the Minimate TFF system (Pall Corporation, New York, NY, USA) equipped with a 10 kDa microcapsule. The colloid, thus concentrated and purified, was used as a plasmonic substrate for the analysis of salivary samples.

### 2.3. SERS Analysis of Salivary Samples

The standard solid SERS substrate was obtained by depositing a quantity of 1 μL of 10× concentrated silver nanoparticles on a substrate preheated to 40 °C of CaF_2_. After complete drying of the nanoparticle spot, a drop of ~1 μL of analyte (whole saliva) was poured on it. Salivary sample measurements were carried out using the inVia Reflex Raman confocal multilaser spectrometer (Renishaw™, Wotton-under-Edge, UK).

The spectra were recorded using the 50× objective, the spectral line was 785 nm, the laser power was 1% (1.95 mW at the sample surface), the total exposure time was 20 s at each point, and the range represented was 350–2350 cm^−1^. The measurements were carried out in the coffee rim area at a maximum of 30 μm from the edge. Two maps of 50 points were made for each test, each map containing 25 acquisitions. In the end, the spectra were mediated using the dedicated Wire 4.2 computer platform and then processed with the help of the OriginPro^®^ 2019 software.

## 3. Results

The performance of the present study has made it possible to form a much clearer perspective regarding the body’s feedback following its exposure to radiative trigger factors such as X-rays.

### 3.1. Data on the Batches Included in This Study

Following the centralization of the data, an equal number of 15 CT samples and 15 CBCT samples were taken. During the processing of the samples, it was necessary to exclude three samples from the batch of patients undergoing CT and one sample from the CBCT batch due to their non-compliance. Thus, in the present study, 12 CT samples and 14 CBCT samples were considered.

### 3.2. SERS Substrates Physico-Chemical Characterization

The quality of the colloids obtained was analyzed using UV-Vis absorption spectroscopy (Figure S1). This method allows the determination of the degree of anisotropy of the synthesized nanoparticles, knowing that the shape and number of absorption peaks depend on the type of material, size, and shape of the synthesized nanoparticles. Out of the need to determine the size and shape of colloidal nanoparticles, TEM measurements were also performed (Figure S2). The results regarding the characterization of plasmonic substrates are presented in the Supplementary Materials attached at the end of this manuscript.

### 3.3. SERS Measurement Results

By acquiring the SERS spectra, it was possible to perform the comparative analysis of salivary samples, both pre- and post-radiative, for each radiological technique studied (CT and CBCT). An image of a sample deposited on the silver plasmonic substrate can be observed in Figure S3. The spectra generated from the SERS analysis will be extensively analyzed and interpreted in the following rows for each radiological technique.

It is important to mention that the Raman spectra of the control group of samples referring to those collected before X-ray radiation were rich in protein vibrational bands, but their intensity is very low as compared to the SERS one (Figures S4 and S5). In this regard, we have chosen to perform SERS measurements to gain more insights into the molecular composition modifications that occur after irradiation. Silver plasmonic substrates’ spectra, as shown in Figures S4 and S5, did not interfere with the salivary samples’ spectra.

#### 3.3.1. SERS Measurements of CT Batch

**Figure 1:**
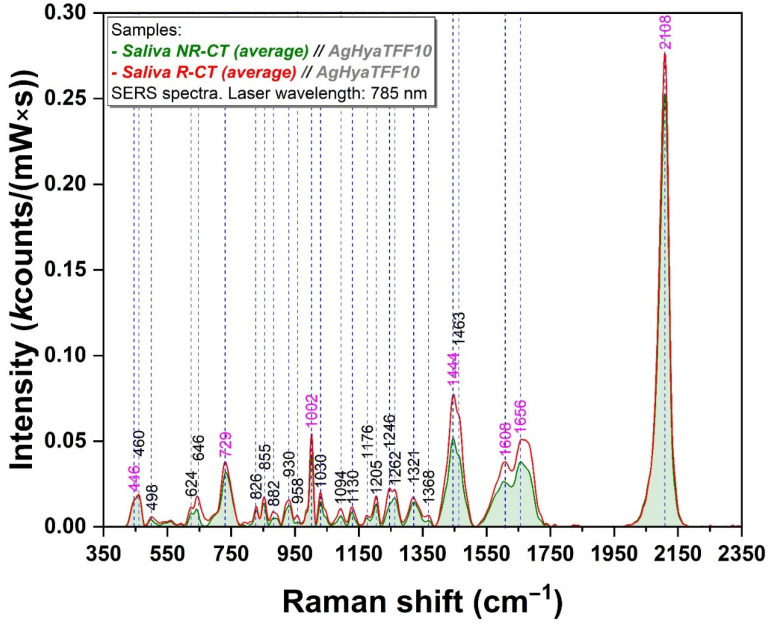
Mean SERS spectra of the CT batch before and after irradiation (green and red spectra represent the average of the analysis of the 12 samples), measured at a laser wavelength of 785 nm. The spectra represent the average of the 12 samples that were previously incubated with the substrate. NR- non-radiation and R- radiation. The magenta-colored peaks represent the most relevant ones that have been amplified after radiation. Refer to Table S1 for details of tentative band assignment.

The spectral analysis of the 12 salivary samples belonging to the CT group is illustrated in Figure 1. The mean spectra of the salivary samples recorded before and after irradiation are highlighted in green and red, respectively (Figure 1). The individual spectra collected from each group of samples are represented in Figures S6 and S7.

Overall, there is a significant increase in all vibrational bands present in the spectra of the samples measured post-irradiation as compared to the pre-irradiation ones. In the case of some bands (729, 1002, 1444, 1463, 1608, 1656, and 2108 cm^−1^), this variation is more visible. Given that these bands have been assigned to the presence of specific biomolecules that have the potential to be considered salivary biomarkers (Table S1) for a broad range of pathologies, the intensities of these bands have been analyzed and compared for each single sample.

#### 3.3.2. SERS Measurement of CBCT Batch

**Figure 2:**
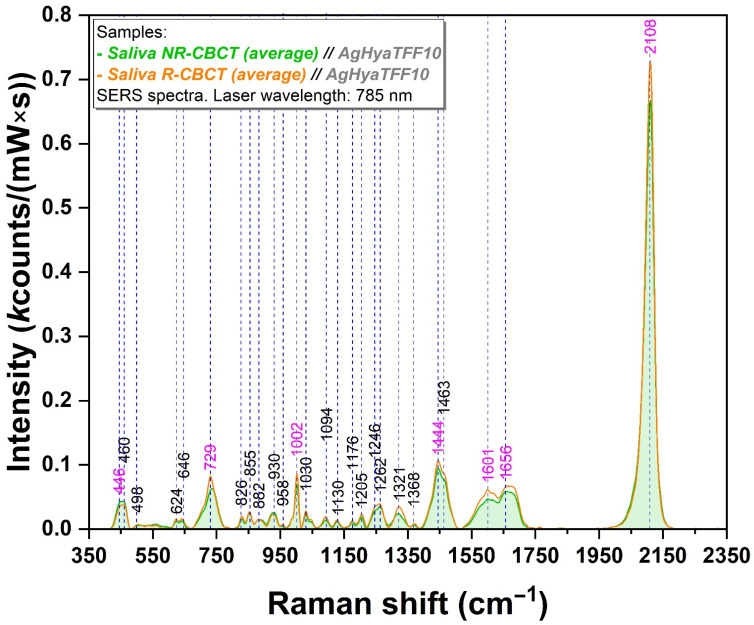
Mean SERS spectra of the CBCT batch before and after irradiation (green and orange spectra represent the average of the analysis of the 14 samples), measured at a laser wavelength of 785 nm. The spectra represent the average of the 14 samples that were previously deposited on the substrate. The magenta-colored peaks represent the most relevant ones that have been amplified after radiation. Refer to Table S1 for details of tentative band assignment.

The spectral analysis of the 14 salivary samples belonging to the CBCT group is illustrated in Figure 2. The mean spectra of the salivary samples recorded before and after irradiation are highlighted in green and orange, respectively (Figure 2). The individual spectra collected from each group of samples are represented in Figures S8 and S9.

At a careful examination of the Raman intensities of the above-mentioned vibrational bands, it has been observed that all of them present an intensity increase ranging from 67 to 155% (for CT samples) and 20 to 63% (for CBCT samples). For better understanding of this increase, the exact values of each band are presented in Table 1 together with the band assignments.

The complete assignment of the vibrational bands recorded in the salivary samples employed in this study is shown in Table S1.

## 4. Discussion

Following the interaction of the body with the X-ray beam employed in CT and CBCT analysis, the following phenomena occur:

- Part of the energy will be stored differently at the tissue level, and this could lead to biochemical repercussions at the molecular level;
- A part of the incident beam will follow an exit path following a disjunct trajectory.

Thus, a phenomenon of energy attenuation of the X-ray beam occurs. As such, the energy difference between the incident and emerging beam is responsible for the occurrence of the radiological image employed in clinical practice [5].

The biological effects of ionizing radiation involve a series of direct and indirect effects that lead to various cytological and systemic changes. They can persist over time and can be transmitted from one generation to another [2,38]. In the case of direct effects, biological molecules absorb energy very quickly, resulting in unstable free radicals, which then regroup into stable forms different from the original molecules. As for the indirect effects of radiation, they occur at the level of water molecules, causing the phenomenon of radiolysis. In this case, the release of unstable free radicals (e.g., hydrogen and hydroxyl ions) can interact with various macromolecules. These unstable radicals can then regroup to form stable molecules. However, these molecules are different in terms of chemical and biochemical properties from the original molecules [5]. The direct and indirect effects of radiation occur in a very narrow time window (~10^−5^ s), but the resulting changes can occur in a few hours or even months [2]. Nevertheless, it could take even more time until these changes can be noticed.

Cellular changes occur in all cell compartments, at the level of nucleic acids, proteins, lipids, and carbohydrates. The nucleus is considered to be the most sensitive cell compartment to the action of ionizing radiation [2]. At the DNA level, these changes refer to the damage or even complete destruction of nitrogenous bases, which leads to alterations in the single and double bonds in the DNA structure, as well as to the impairment of DNA–DNA and DNA–protein interactions. These will cause various mutations at the cellular level depending on the phases of mitotic cell division [2,5].

The cells in the oral cavity come into direct contact with saliva, and it is suspected that the following biomarkers could also be analyzed from a saliva sample, demonstrating the relevance of this biofluid in medical investigations.

Current studies investigating the influence of X-ray exposure analyzed in terms of salivary secretion in rats, puppies, and lambs indicate a possible change in saliva flow and composition, associated with an increase in potassium ion levels [39]. However, further studies should be performed for a better understanding concerning these complex phenomena.

The present study manages to highlight the compositional changes in the salivary fluid following patients’ exposure to X-rays from the perspective of a vibrational spectroscopic analysis. This approach is not very common in the scientific literature. At the same time, the promising results obtained are a novelty in the field and provide the premises for future studies to deepen this theme due to its valuable but insufficiently exploited applicative potential. In order to emphasize the above, we recall the results of the research carried out by our group in which we had a more simplistic approach to the topic of assessing the effects of Röntgen radiation on the oral cavity [20].

By performing a comparative analysis of the acquired Raman versus SERS spectra, it is worth mentioning that there is a significant abundance of protein vibrational bands in the case of Raman ones (Figures S4 and S5). In order to evaluate the oxidative stress caused by the exposure of the oral cavity to Röntgen radiation, it was necessary to quantify some biomolecules with a potential marker role. One of the major impediments in achieving this is represented by the significantly lower concentration of these biomolecules compared to the major protein component present at the level of the liquid biopsies analyzed. This is the reason why the analysis of salivary samples by means of SERS was also included in this study.

The use of a solid plasmonic substrate for SERS analysis was preferred to the incubation of salivary samples with plasmonic nanoparticles. The rationale was to prevent the occurrence of complications such as the irreproducible formation of plasmonic hot spots leading to different types of nanoscale interaction between biomolecules and plasmonic nanoparticles, which in turn would result in serious disturbance of the signals of the molecules of interest and a lack of spectral reproducibility.

In the case of the group exposed to the CT radiological technique, an overall increase in the vibrational bands corresponding to the post-radiative context is observed (Figure 1). At the same time, an identical tropism can be noted in the case of CBCT (Figure 2).

Without making a proper comparison between the two imaging techniques investigated, it can be mentioned that there is a greater discrepancy in the case of the CT technique between the non-radiative and radiative states (Figure 1). In both cases, the most important vibrational bands are located at 446, 729, 1002, 1444, 1446, 1601, 1608, 1656, and 2108 cm^−1^ (Figure 1 and Figure 2, Table 1). These bands have been assigned to molecules with potential roles as biomarkers for oral, maxillofacial, and systemic pathologies [6,7,20].

The spectrum is dominated by thiocyanate, which has three characteristic peaks located at 446, 729, and 2108 cm^−1^ [20,21,22] the latter being the most intense one. At the level of the oral cavity, thiocyanate has different roles, as an antimicrobial and an antioxidant, its concentration being directly proportional to the oxidative stress status. Moreover, thiocyanate can represent an indirect indicator of cytotoxicity already existing at the intracellular and intercellular level. Radiation-induced stress has turned into the amplification of thiocyanate-specific vibrational bands. Thiocyanate has the property of neutralizing the cyanate formed by the decomposition of nitrogen-containing radicals under X-ray exposure. It has the ability to bind to metallic surfaces such as silver and gold nanoparticles used in SERS applications, involving N-C or S-C bonds and different vibrational modes [14,40,41].

Opiorphin, representing another molecule of interest, stands out for its analgesic and antioxidant properties. Its properties have been studied for a long time, and there are numerous articles on the subject [7,42,43]. The vibrational band associated with it is the one at 1002 cm^−1^ [7], being more intense in the post-radiative analyzed batches in both CT and CBCT. Physiological stressors such as radiation exposure might influence its secretion as part of the body’s pain regulator.

The whole spectrum abounds in protein- and amino-acid-specific vibrational bands (460, 624, 855, 882, 930, 958, 1030, 1130, 1176, 1205, 1246, 1262, 1321, 1368, 1463, 1601/1608, and 1656 cm^−1^) (Table S1). Phenylalanine, represented by the 1601 and 1608 cm^−1^ vibrational bands, stands out in the post-irradiation spectra. Amide I, found at 1656 cm^−1^, can reveal structural damage in proteins caused by ionizing radiation [23]. Mucin, associated with 1444/1446 cm^−1^ vibrational bands, is another major representative of the organic matter present in saliva [21,26,28,30,31,44].

The discrepancies between the two types of techniques studied in the present paper leave room for future research aiming to investigate the biochemical differences at the molecular level between them. Moreover, the inclusion of a larger number of samples will provide new insights into the effects of X-rays on the composition of salivary secretion.

## 5. Conclusions

SERS spectroscopy retains its established usefulness for the study of molecular dynamics at the nanoscale. Silver-based plasmonic substrates have a remarkable qualitative and quantitative discriminating capacity in the study of compositional variations in the samples to be investigated. The use of a different method of analysis of the liquid biopsies offered a different but complementary perspective on the interaction of the molecules of interest with the plasmonic substrates used.

Despite the protein dominance of the acquired spectra, the clinical relevance of the results obtained remained unscathed, an aspect that greatly simplifies the future methodologies that will underpin the development of affordable diagnostic platforms at low costs.

A broader perspective on the study of the biological effects of X-rays could be obtained by evaluating the actual differences between the two imaging techniques under discussion. It is widely known that in the case of CBCT, the irradiation dose is lower compared to CT.

The present study proves that SERS can be successfully used in the detection of molecular changes in saliva as a consequence of direct interaction between X-rays and the oral cavity. This conclusion is further supported by our findings: the increase in the intensities of the bands associated with thiocyanate and opiorphin is much higher in the case of CT as compared to CBCT. This demonstrates once again the roles of thiocyanate and opiorphin as potential salivary biomarkers. SERS demonstrates its ability to detect changes in salivary thiocyanate and opiorphin levels occurring in a very short time period. Thus, perspectives are opened for developing new applications.

## Figures and Tables

**Table 1:** Band assignment and post-irradiation intensity increase values of the proposed spectral bands observed after CT and CBCT investigations, respectively.

Vibrational Band (cm^−1^)	Assignment	Post-Irradiation Increase in Band Intensities After CT (%)	Post-Irradiation Increase in Band Intensities After CBCT (%)
**446**	Thiocyanate, phenylalanine [20,21,22]	100 ± 1.9	25 ± 0.6
**729**	Hypoxanthine, tryptophan, coenzyme A, nucleic acids, thiocyanate [20,21,22,23,24,25]	77 ± 1.8	49 ± 0.8
**1002**	L-Phenylalanine, uric acid, opiorphin, pyranose [7,20,26,27,28,29]	95 ± 1.3	21 ± 0.6
**1444/1446**	Mucin matrix, collagen, phospholipids [21,26,28,30,31]	129 ± 1.8	38 ± 0.9
**1601/1608**	Phenylalanine, adenine [32,33,34]	154 ± 2.1	63 ± 1.4
**1656**	Amide I, nucleic acids, glutathione [20,23,30,35,36,37]	129 ± 2.1	47 ± 1.1
**2108**	Thiocyanate [14,20,22]	67 ± 1.2	26 ± 0.4

## Data Availability

All details regarding patients’ database and many more significant information can be found in Supplementary Materials Files. If more information is needed, please contact the corresponding authors.

## Supplementary Materials

The following supporting information can be downloaded at: https://www.mdpi.com/article/10.3390/s24248021/s1, Figure S1: UV-Vis absorption spectrum of Ag nanoparticles. The inset represents a photograph of the silver colloid after TFF concentration. Figure S2: Size distribution of Ag nanoparticles together with a TEM image in the right corner of the figure. Figure S3: Optical image of a saliva sample deposited on solid silver plasmonic substrate. Figure S4: Mean Raman spectra (olive) of control saliva samples measured at a laser wavelength of 785 nm together with SERS spectra of the CT batch before irradiation (green) and AgHyaTFF10 spectra (blue) measured at a laser wavelength of 785 nm. Figure S5: Mean Raman spectra (olive) of control saliva samples measured at a laser wavelength of 785 nm together with SERS spectra of the CBCT batch before irradiation (grass green) and AgHyaTFF10 spectra (blue) measured at a laser wavelength of 785 nm. Figure S6: Individual SERS spectra recorded on salivary samples collected before CT investigation. The green spectrum represents the average of the analysis of the 12 samples, while the grey spectra correspond to each sample. Figure S7: Individual SERS spectra recorded on salivary samples collected after CT investigation. The red spectrum represents the average of the analysis of the 12 samples, while the grey spectra correspond to each sample. Figure S8: Individual SERS spectra recorded on salivary samples collected before CBCT investigation. The green spectrum represents the average of the analysis of the 14 samples, while the grey spectra correspond to each sample. Figure S9: Individual SERS spectra recorded on salivary samples collected after CBCT investigation. The orange spectrum represents the average of the analysis of the 14 samples, while the grey spectra correspond to each sample. Table S1: Tentative assignment of vibrational band. Refs. [7,14,20,21,22,23,24,25,26,27,28,29,30,31,32,33,34,35,36,37,45,46,47,48,49,50] are cited in Supplementary Materials files.
