# Supplementary material for: Understanding the Interaction of Röntgen Radiation Employed in Computed Tomography/Cone Beam Computed Tomography Investigations of the Oral Cavity by Means of Surface-Enhanced Raman Spectroscopy Analysis of Saliva
Source: Sensors (Basel). 2024 Dec 16;24(24):8021. doi: 10.3390/s24248021 (PMC11679455; doi:10.3390/s24248021)
Supplement: Supplementary file 1 [file sensors-24-08021-s001.zip › sensors-3244473-supplementary.pdf]

Supplementary Files

Understanding the Interaction of Röntgen Radiation Employed in Computed Tomography/Cone Beam Computed Tomography Investigations of the Oral Cavity by Means of Surface-Enhanced Raman Spectroscopy Analysis of Saliva

Rareș-Mario Borșa [1,2,3,4,5,†], Valentin Toma [5,†], Melania-Teodora Nășcuțiu [1,5], Anca Onaciu [3,5], Ioana-Maria Colceriu-Șimon [6], Grigore Băciuț [1,2,7], Simion Bran [1,2,7], Cristian-Mihail Dinu [1,2,7], Florin Onișor [1,2,7], Gabriel Armencea [1,2,7], Carina Culic [8], Mihaela-Carmen Hedeșiu [7,9], Rareș-Ionuț Știufiuc [3,5,10,*] and Mihaela-Felicia Băciuț [1,2,7]

[1] Dental Medicine Faculty, "Iuliu Hatieganu" University of Medicine and Pharmacy, Pasteur 4,
400349 Cluj-Napoca, Romania; rares.mari.borsa@elearn.umfcluj.ro (R.-M.B.); melania.teod.nascutiu@elearn.umfcluj.ro (M.-T.N.); gbaciut@umfcluj.ro (G.B.); dr_brans@umfcluj.ro (S.B.); cristian.dinu@umfcluj.ro (C.-M.D.); florin.onisor@umfcluj.ro (F.O.); armencea.gabriel@umfcluj.ro (G.A.); mbaciut@umfcluj.ro (M.-F.B.)

[2] Department of Maxillofacial Surgery and Implantology, "Iuliu Hațieganu" University of Medicine and Pharmacy, Cardinal Iuliu Hossu 37, 400029 Cluj-Napoca, Romania

[3] Department of Pharmaceutical Physics & Biophysics, Faculty of Pharmacy, "Iuliu Hatieganu" University of Medicine and Pharmacy, Louis Pasteur 6, 400349 Cluj-Napoca, Romania; anca.onaciu@umfcluj.ro

[4] Department of Prosthetic Dentistry and Dental Materials, Division Dental Propaedeutics, Aesthetics, Dental Medicine Faculty, "Iuliu Hatieganu" University of Medicine and Pharmacy, Clinicilor 32,
400001 Cluj-Napoca, Romania

[5] Department of NanoBioPhysics, Institute of Medical Research and Life Sciences—MEDFUTURE, "Iuliu Haţieganu" University of Medicine and Pharmacy, Louis Pasteur 4-6, 400349 Cluj-Napoca, Romania;
valentin.toma@umfcluj.ro

[6] Department of Conservative Odontology, Division Orthodontics and Dental-Facial Orthopedics, "Iuliu
Hatieganu" University of Medicine and Pharmacy, Avram Iancu 31, 400089 Cluj-Napoca, Romania;
simon.ioana@umfcluj.ro

[7] County Emergency Hospital Cluj, Clinicilor 3-5, 400006 Cluj-Napoca, Romania; mhedesiu@umfcluj.ro

[8] Department of Conservative Odontology, Division Odontology, Endodontics, Cariology, Oral Pathology, "Iuliu Hatieganu" University of Medicine and Pharmacy, Moților 33, 400089 Cluj-Napoca, Romania;
culic@elearn.umfcluj.ro

[9] Department of Oral Radiology, "Iuliu Hatieganu" University of Medicine and Pharmacy, Avram Iancu 31, 400089 Cluj-Napoca, Romania

[10] Nanotechnology Laboratory, TRANSCEND Research Center, Regional Institute of Oncology, General Henri Mathias Berthelot 2-4, 700483 Iaşi, Romania

* Correspondence: rares.stiufiuc@umfcluj.ro; Tel.: +40-726340278

† These authors contributed equally to this work.

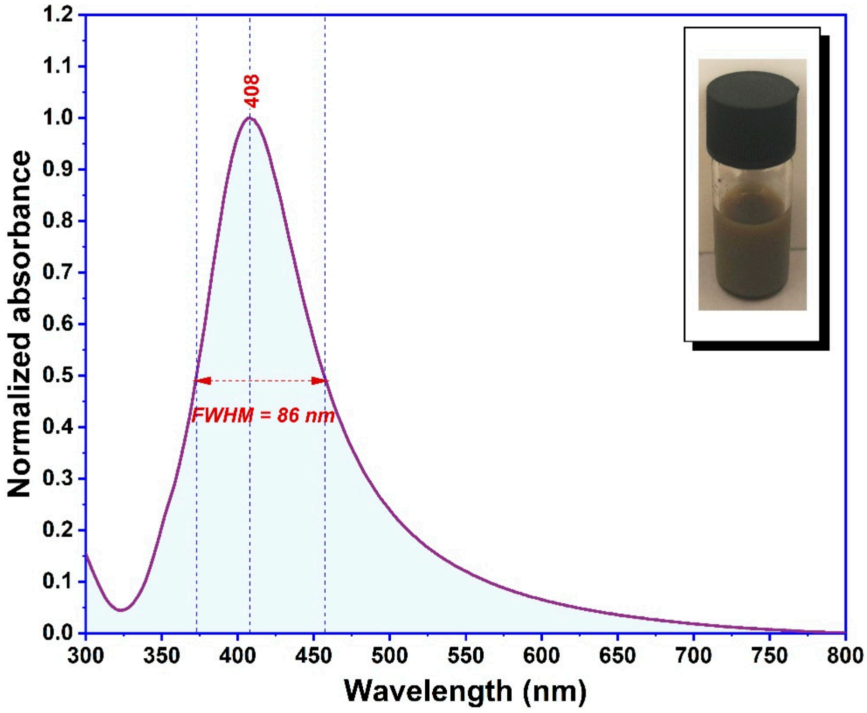

Figure S1: UV-Vis absorption spectrum of Ag nanoparticles. The inset represents a photograph of the silver colloid after TFF concentration.

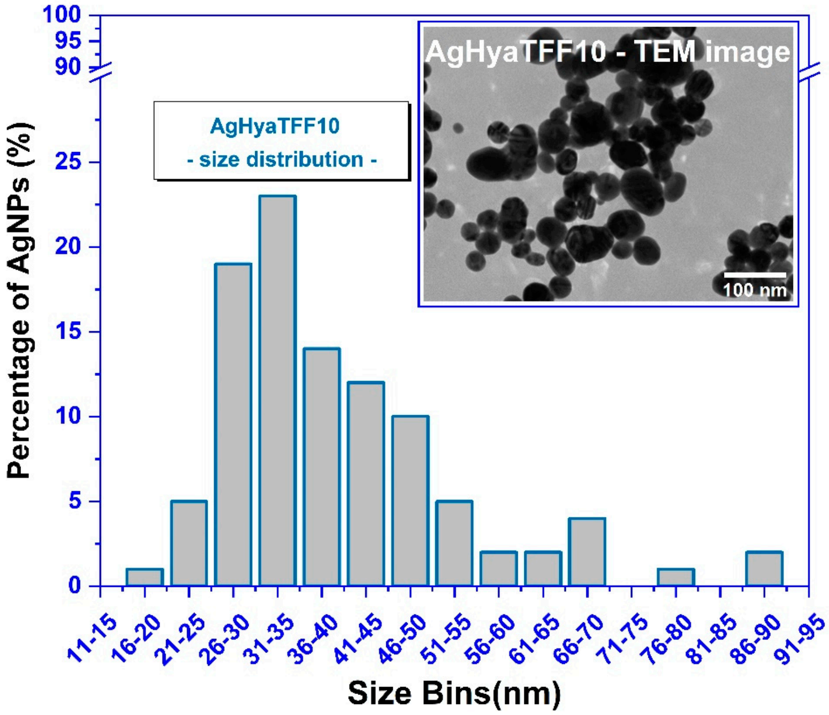

Figure S2: Size distribution of Ag nanoparticles together with a TEM image in the right corner of the figure.

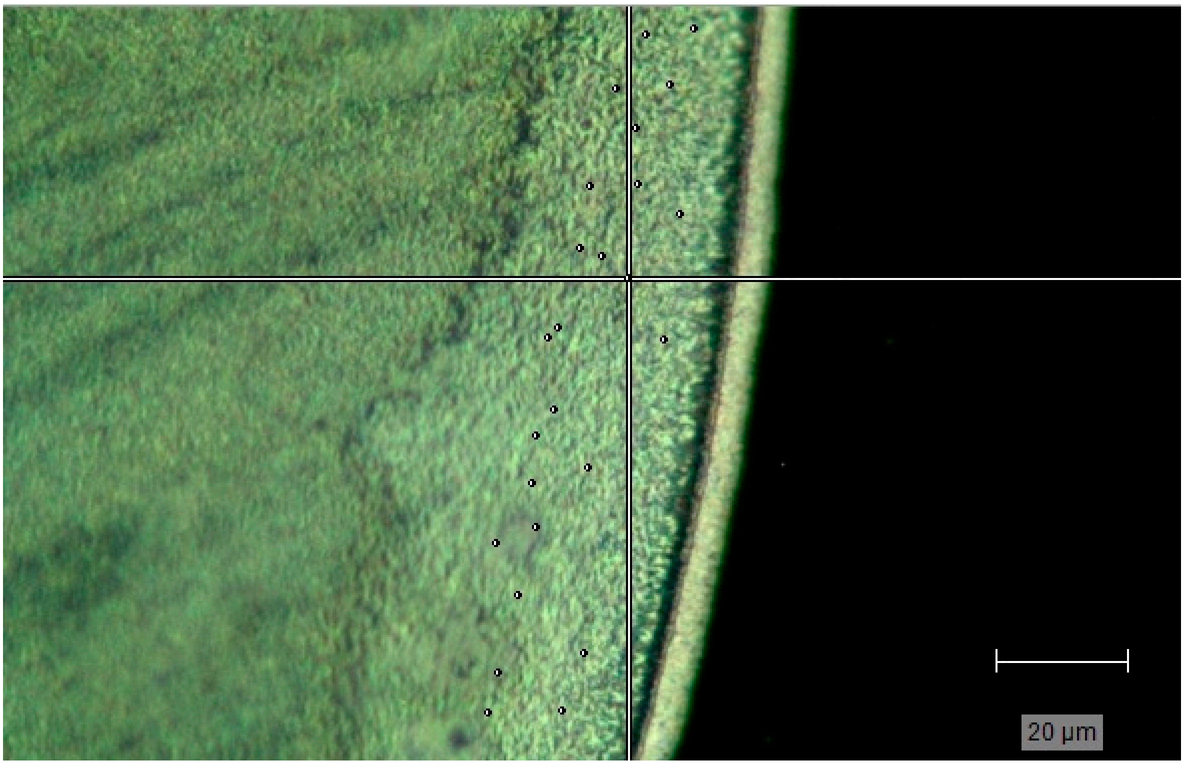

Figure S3: Optical image of a saliva sample deposited on solid silver plasmonic substrate.

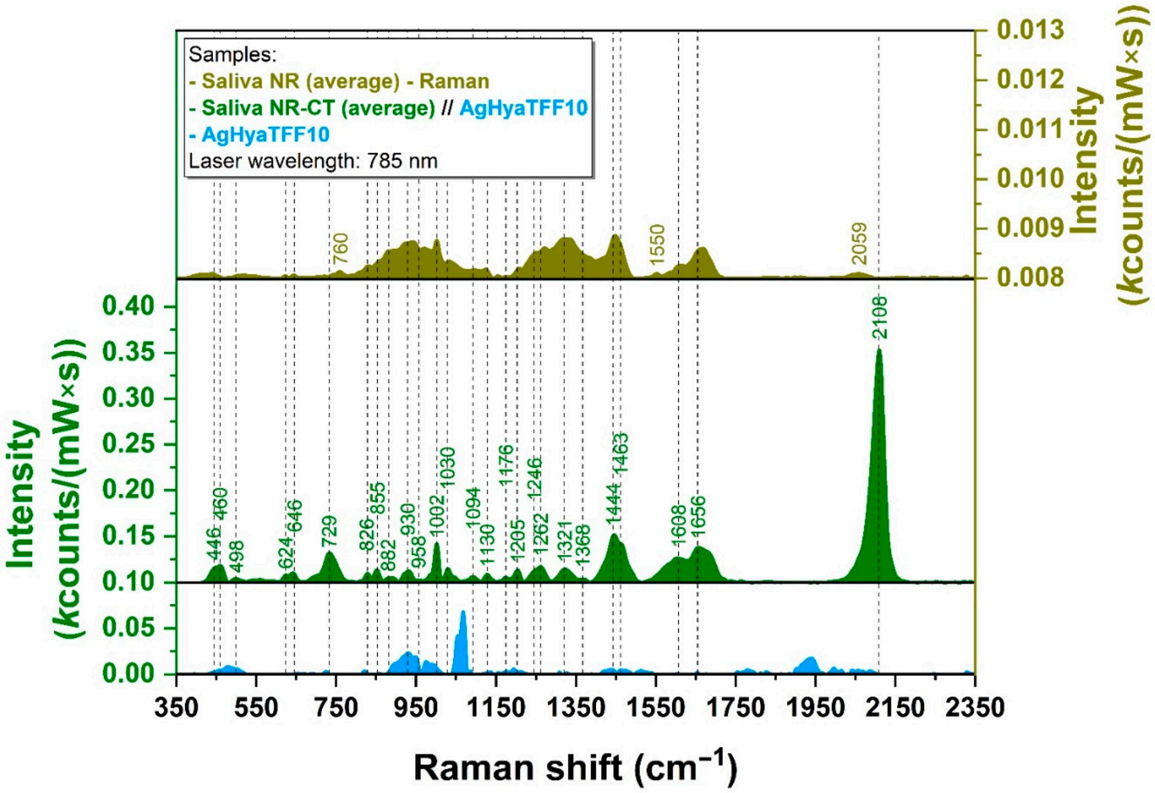

Figure S4: Mean Raman spectra (olive) of control saliva samples measured at a laser wavelength of 785 nm together with SERS spectra of the CT batch before irradiation (green) and AgHyaTFF10 spectra (blue) measured at a laser wavelength of 785 nm.

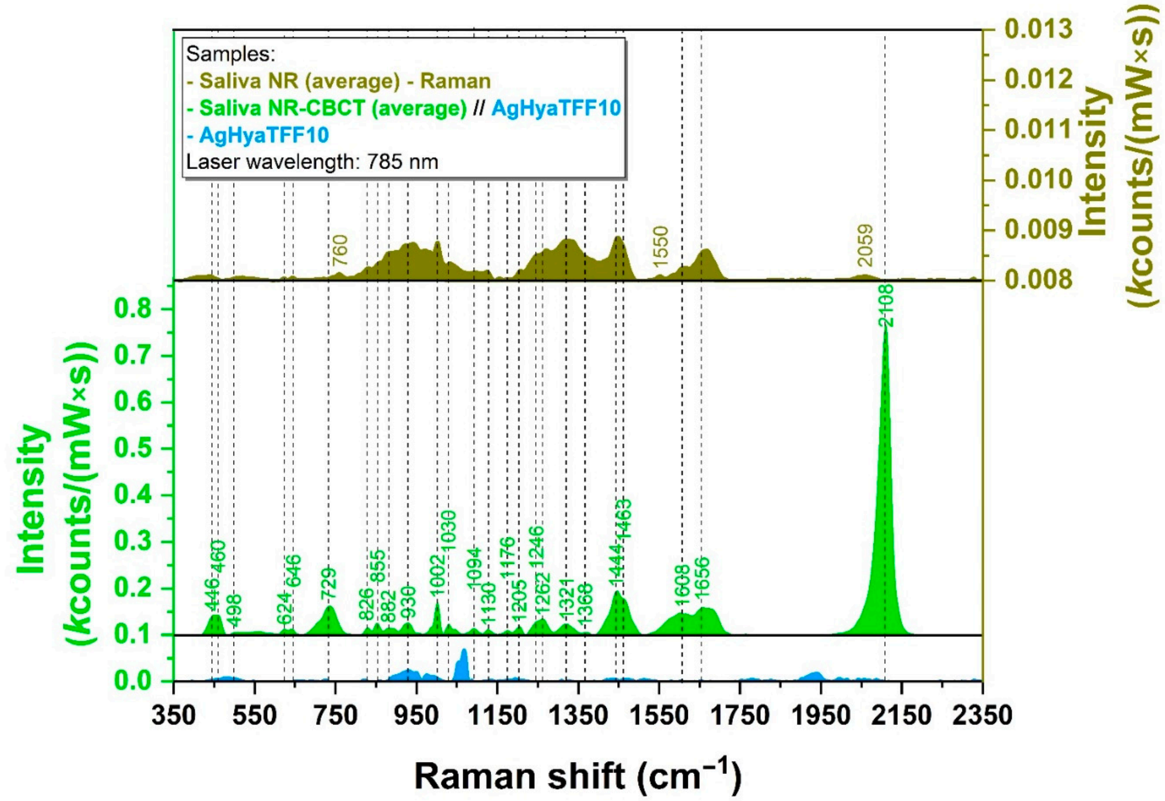

Figure S5: Mean Raman spectra (olive) of control saliva samples measured at a laser wavelength of 785 nm together with SERS spectra of the CBCT batch before irradiation (grass green) and AgHyaTFF10 spectra (blue) measured at a laser wavelength of 785 nm.

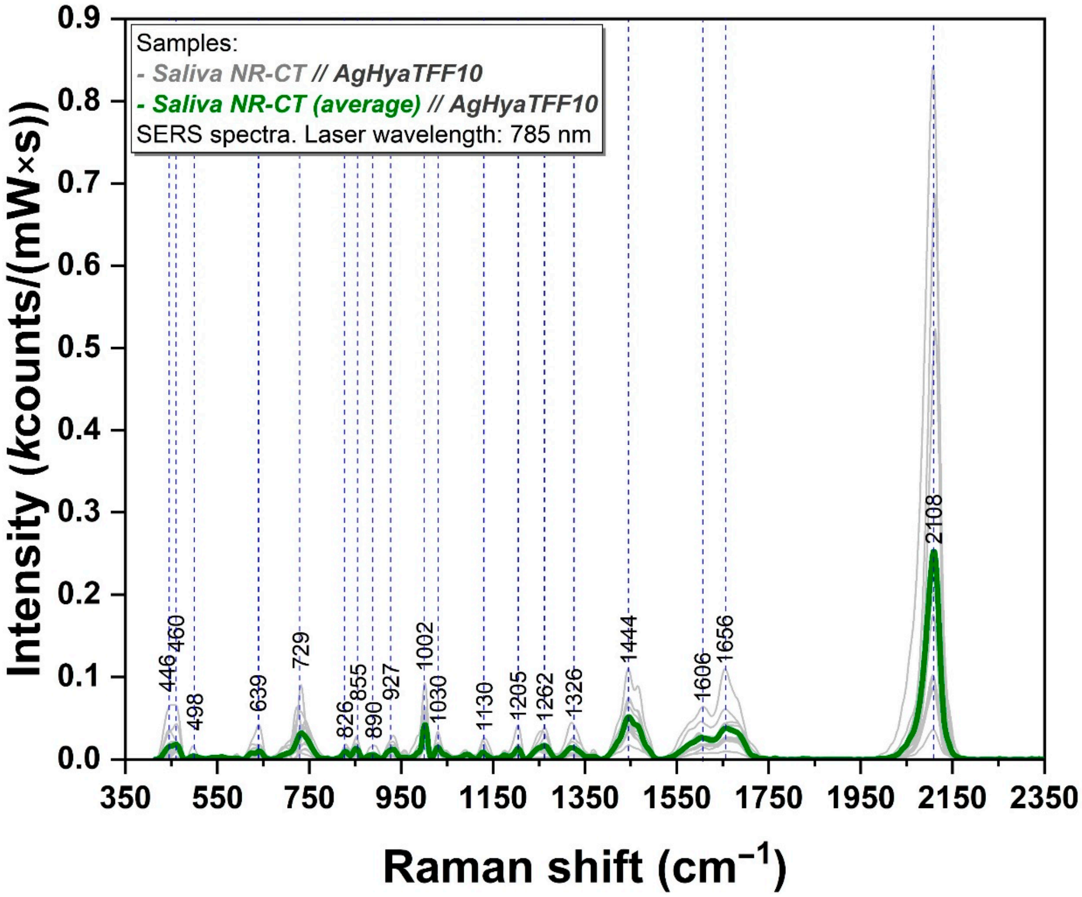

Figure S6: Individual SERS spectra recorded on salivary samples collected before CT investigation. The green spectrum represents the average of the analysis of the 12 samples, while the grey spectra correspond to each sample.

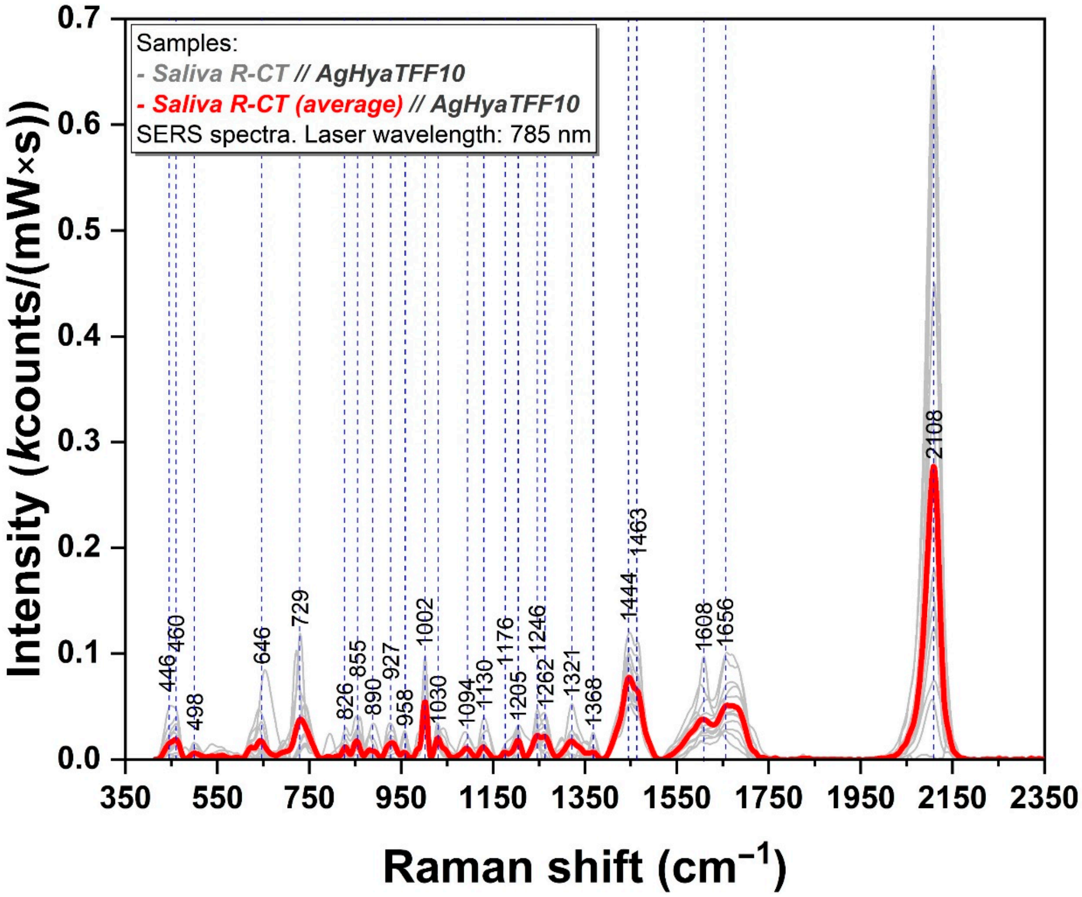

Figure S7: Individual SERS spectra recorded on salivary samples collected after CT investigation. The red spectrum represents the average of the analysis of the 12 samples, while the grey spectra correspond to each sample.

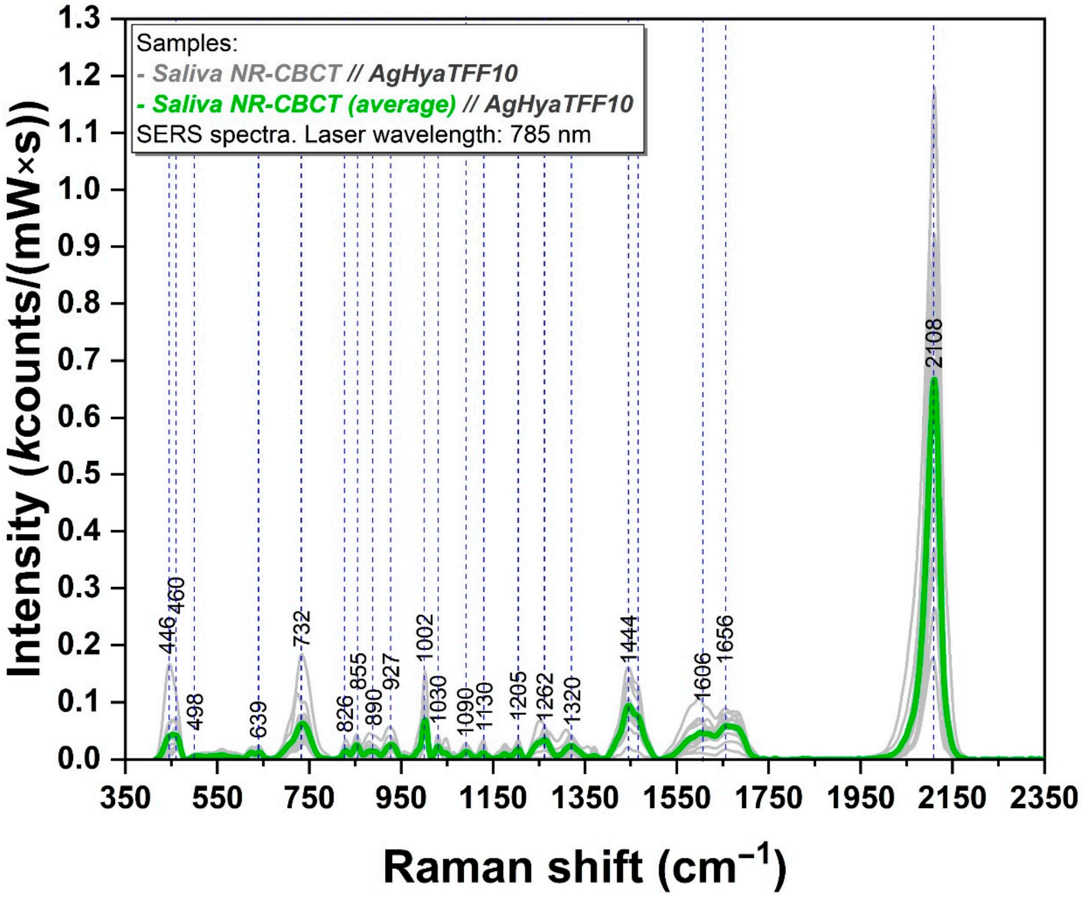

Figure S8: Individual SERS spectra recorded on salivary samples collected before CBCT investigation. The green spectrum represents the average of the analysis of the 14 samples, while the grey spectra correspond to each sample.

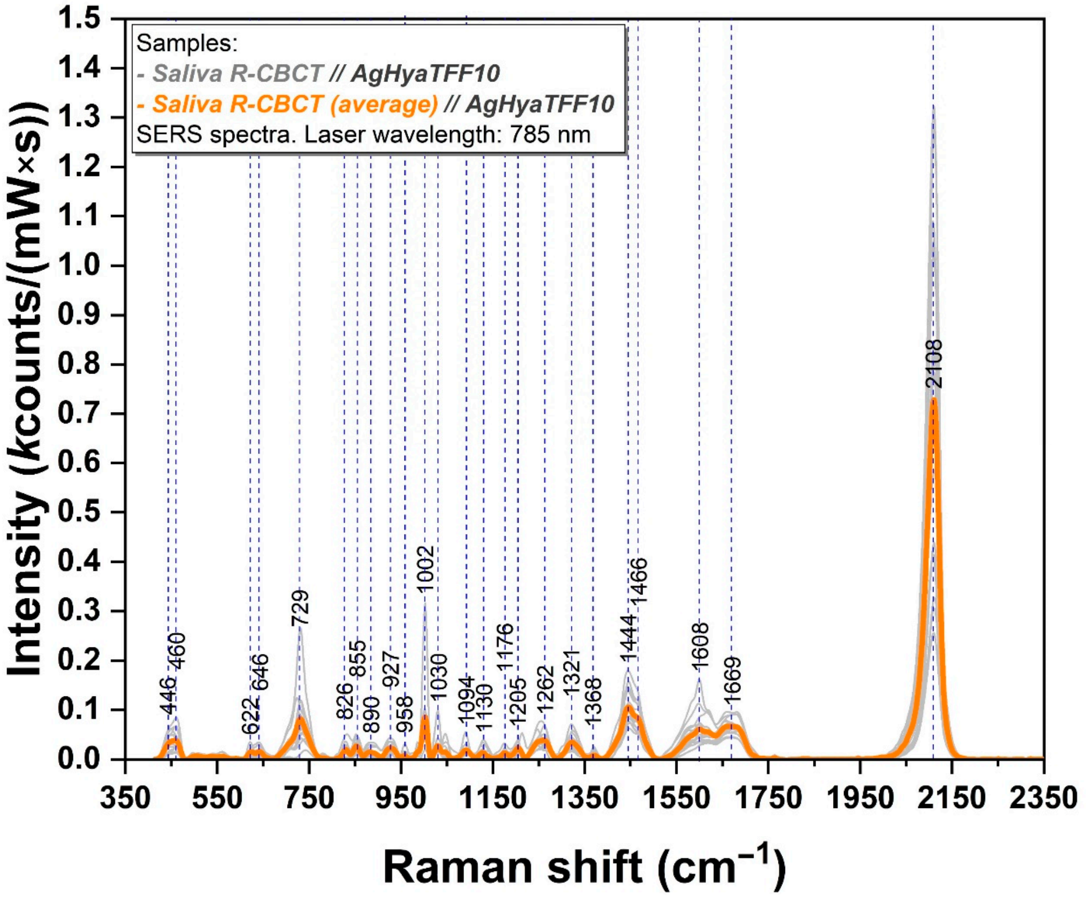

Figure S9: Individual SERS spectra recorded on salivary samples collected after CBCT investigation. The orange spectrum represents the average of the analysis of the 14 samples, while the grey spectra correspond to each sample.

Table S1: Tentative assignment of vibrational bands

Vibrational band (cm^{-1})	Molecule	References
446	Thiocyanate, phenylalanine	[1–3]
460	L-Tryptophan, nucleic acids, saccharides	[4]
498	Uric acid, polysaccharide, glycogen	[2,5]
624	Proteins, phenylalanine, adenine	[1,6]
646	Phenylalanine, tyrosine	[1,7,8]
729/732	Hypoxanthine, tryptophan, coenzyme A, nucleic acids, thiocyanate	[1–3,6,9,10]
826	Glutathione	[11]
855	L-Phenylananine, L-Proline, tyrosine	[8,12]
882	Tryptophan, proline, valine,	[1,6,8,13,14]

	glycine, glutamic acid, hydroxyprolin, uric acid	
930	Proteins, glucose, proline, valine	[6–8,11,15–17]
958	Proline, valine, xanthine	[8,13]
1002	L-Phenylananine, uric acid, opiorphin, pyranose	[1,7,14,17–19]
1030	Phenylalanine	[7,8,20]
1094	Azelaic acid, 3-phosphoglycerate, 1-monopalmitin, hypoxanthine	[13,14]
1130	L-Tyrosine, histidine, uric acid, lactic acid, D-mannose, xanthine	[2,11,13]
1176	Proteins, lipids, phenylalanine, tyrosine	[7,8,11,14]
1205	L-tyrosine, L-tryptophan, phenylalanine, proteins	[1,2,8]
1246	Amide III, xanthine	[12,13]
1262	Tyrosine, cytosine, guanine, adenine , amide III, collagen	[8,11]
1321	L-tyrosine, L-tryptophan, nucleic acids, hypoxanthine	[13,21]
1368	Lipids, tryptophan, porphyrins, guanine, thymine, proteins, uric acid	[6,12,13]
1444/1446	Mucin matrix, collagen, phospholipids	[2,7,8,12,14]
1463	Amide I	[11]
1601/1608	Phenylalanine, adenine	[15,22,23]
1656	Amide I, nucleic acids, glutathione	[1,9,11,12,24,25]
2108	Thiocyanate	[1,3,26]

References

1. Colceriu-Șimon; Hedeșiu; Toma; Armencea; Moldovan; Știufiuc; Culic; Țărmure; Dinu; Berindan-Neagoe; et al. The Effects of Low-Dose Irradiation on Human Saliva: A Surface-Enhanced Raman Spectroscopy Study. *Diagnostics* **2019**, *9*, 101, doi:10.3390/diagnostics9030101.
2. Qiu, S.; Xu, Y.; Huang, L.; Zheng, W.; Huang, C.; Huang, S.; Lin, J.; Lin, D.; Feng, S.; Chen, R.; et al. Non-Invasive Detection of Nasopharyngeal Carcinoma Using Saliva Surface-Enhanced Raman Spectroscopy. *Oncol. Lett.* **2016**, *11*, 884–890, doi:10.3892/ol.2015.3969.
3. Wong, M. Surface-Enhanced Raman Spectroscopy for Forensic Analysis of Human Saliva, Boston University, 2017.
4. Calado, G.; Behl, I.; Byrne, H.J.; Lyng, F.M. Raman Spectroscopic Characterisation of Non Stimulated and Stimulated Human Whole Saliva. *Clin. Spectrosc.* **2021**, *3*, 100010, doi:10.1016/j.clispe.2021.100010.
5. Kho, K.W.; Fu, C.Y.; Dinish, U.S.; Olivo, M. Clinical SERS: Are We There Yet? *J. Biophotonics* **2011**, *4*, 667–684, doi:10.1002/jbio.201100047.
6. Li, X.; Yang, T.; Lin, J. Spectral Analysis of Human Saliva for Detection of Lung Cancer Using

Surface-Enhanced Raman Spectroscopy. *J. Biomed. Opt.* **2012**, *17*, 037003, doi:10.1117/1.jbo.17.3.037003.
7. Feng, S.; Lin, D.; Lin, J.; Huang, Z.; Chen, G.; Li, Y.; Huang, S.; Zhao, J.; Chen, R.; Zeng, H. Saliva Analysis Combining Membrane Protein Purification with Surface-Enhanced Raman Spectroscopy for Nasopharyngeal Cancer Detection. *Appl. Phys. Lett.* **2014**, *104*, doi:10.1063/1.4866027.
8. Lin, X.; Lin, D.; Ge, X.; Qiu, S.; Feng, S.; Chen, R. Noninvasive Detection of Nasopharyngeal Carcinoma Based on Saliva Proteins Using Surface-Enhanced Raman Spectroscopy. *J. Biomed. Opt.* **2017**, *22*, 1, doi:10.1117/1.JBO.22.10.105004.
9. Derruau, S.; Robinet, J.; Untereiner, V.; Piot, O.; Sockalingum, G.D.; Lorimier, S. Vibrational Spectroscopy Saliva Profiling as Biometric Tool for Disease Diagnostics: A Systematic Literature Review. *Molecules* **2020**, *25*, 4142, doi:10.3390/molecules25184142.
10. Ma, L.; Zhang, Z.; Li, X. Non-Invasive Disease Diagnosis Using Surface-Enhanced Raman Spectroscopy of Urine and Saliva. *Appl. Spectrosc. Rev.* **2020**, *55*, 197–219, doi:10.1080/05704928.2018.1562938.
11. Rekha, P.; Aruna, P.; Brindha, E.; Koteeswaran, D.; Baludavid, M.; Ganesan, S. Near-infrared Raman Spectroscopic Characterization of Salivary Metabolites in the Discrimination of Normal from Oral Premalignant and Malignant Conditions. *J. Raman Spectrosc.* **2016**, *47*, 763–772, doi:10.1002/jrs.4897.
12. Meenapriya, P. Raman Spectroscopic Analysis of Blood, Urine, Saliva and Tissue of Oral Potentially Malignant Disorders and Malignancy-A Diagnostic Study. *Int. J. Oral Craniofacial Sci.* **2016**, 011–014, doi:10.17352/2455-4634.000013.
13. Stefancu, A.; Badarinza, M.; Moisoiu, V.; Iancu, S.D.; Serban, O.; Leopold, N.; Fodor, D. SERS-Based Liquid Biopsy of Saliva and Serum from Patients with Sjögren's Syndrome. *Anal. Bioanal. Chem.* **2019**, *411*, 5877–5883, doi:10.1007/s00216-019-01969-x.
14. Koster, H.J.; Guillen-Perez, A.; Gomez-Diaz, J.S.; Navas-Moreno, M.; Birkeland, A.C.; Carney, R.P. Fused Raman Spectroscopic Analysis of Blood and Saliva Delivers High Accuracy for Head and Neck Cancer Diagnostics. *Sci. Rep.* **2022**, *12*, 1–13, doi:10.1038/s41598-022-22197-x.
15. Lee, C.; Carney, R.P.; Hazari, S.; Smith, Z.J.; Knudson, A.; Robertson, C.S.; Lam, K.S.; Wachsmann-Hogiu, S. 3D Plasmonic Nanobowl Platform for the Study of Exosomes in Solution. *Nanoscale* **2015**, *7*, 9290–9297, doi:10.1039/C5NR01333J.
16. Zhang, H.; Silva, A.C.; Zhang, W.; Rutigliano, H.; Zhou, A. Raman Spectroscopy Characterization Extracellular Vesicles from Bovine Placenta and Peripheral Blood Mononuclear Cells. *PLoS One* **2020**, *15*, e0235214, doi:10.1371/JOURNAL.PONE.0235214.
17. Buchan, E.; Kelleher, L.; Clancy, M.; Stanley Rickard, J.J.; Oppenheimer, P.G. Spectroscopic Molecular-Fingerprint Profiling of Saliva. *Anal. Chim. Acta* **2021**, *1185*, 339074, doi:10.1016/j.aca.2021.339074.
18. Borșa, R.-M.; Toma, V.; Onaciu, A.; Moldovan, C.-S.; Mărginean, R.; Cenariu, D.; Științiuc, G.-F.; Dinu, C.-M.; Bran, S.; Opriș, H.-O.; et al. Developing New Diagnostic Tools Based on SERS Analysis of Filtered Salivary Samples for Oral Cancer Detection. *Int. J. Mol. Sci.* **2023**, *24*, 12125, doi:10.3390/ijms241512125.
19. Hernández-Arteaga, A.; de Jesús Zermeño Nava, J.; Kolosovas-Machuca, E.S.; Velázquez-Salazar, J.J.; Vinogradova, E.; José-Yacamán, M.; Navarro-Contreras, H.R. Diagnosis of Breast Cancer by Analysis of Sialic Acid Concentrations in Human Saliva by Surface-Enhanced Raman Spectroscopy of Silver Nanoparticles. *Nano Res.* **2017**, *10*, 3662–3670, doi:10.1007/s12274-017-1576-5.
20. Feng, S.; Huang, S.; Lin, D.; Chen, G.; Xu, Y.; Li, Y.; Huang, Z.; Pan, J.; Chen, R.; Zeng, H. Surface-Enhanced Raman Spectroscopy of Saliva Proteins for the Noninvasive Differentiation of Benign and Malignant Breast Tumors. *Int. J. Nanomedicine* **2015**, *10*, 537–547, doi:10.2147/IJN.S71811.
21. Stremersch, S.; Marro, M.; Pinchasik, B. El; Baatsen, P.; Hendrix, A.; De Smedt, S.C.; Loza-Alvarez, P.; Skirtach, A.G.; Raemdonck, K.; Braeckmans, K. Identification of Individual

Exosome-like Vesicles by Surface Enhanced Raman Spectroscopy. *Small* **2016**, *12*, 3292–3301, doi:10.1002/smll.201600393.

22. Maitra, I.; Morais, C.L.M.; Lima, K.M.G.; Ashton, K.M.; Date, R.S.; Martin, F.L. Attenuated Total Reflection Fourier-Transform Infrared Spectral Discrimination in Human Bodily Fluids of Oesophageal Transformation to Adenocarcinoma. *Analyst* **2019**, *144*, 7447–7456, doi:10.1039/C9AN01749F.
23. Maitra, I.; Morais, C.L.M.; Lima, K.M.G.; Ashton, K.M.; Date, R.S.; Martin, F.L. Raman Spectral Discrimination in Human Liquid Biopsies of Oesophageal Transformation to Adenocarcinoma. *J. Biophotonics* **2020**, *13*, doi:10.1002/jbio.201960132.
24. Gonchukov, S.; Sukhinina, A.; Bakhmutov, D.; Minaeva, S. Raman Spectroscopy of Saliva as a Perspective Method for Periodontitis Diagnostics. *Laser Phys. Lett.* **2012**, *9*, 73–77, doi:10.1002/lapl.201110095.
25. Virkler, K.; Lednev, I.K. Forensic Body Fluid Identification: The Raman Spectroscopic Signature of Saliva. *Analyst* **2010**, *135*, 512–517, doi:10.1039/B919393F.
26. Fălămaș, A.; Rotaru, H.; Hedeșiu, M. Surface-Enhanced Raman Spectroscopy (SERS) Investigations of Saliva for Oral Cancer Diagnosis. *Lasers Med. Sci.* **2020**, *35*, 1393–1401, doi:10.1007/s10103-020-02988-2.
